# Lessons learned from community engagement regarding phylodynamic research with molecular HIV surveillance data

**DOI:** 10.1002/jia2.26111

**Published:** 2023-07-06

**Authors:** Diana M. Tordoff, Brian Minalga, Alfredo Trejo, Alic Shook, Roxanne P. Kerani, Joshua T. Herbeck

**Affiliations:** ^1^ Department of Epidemiology University of Washington Seattle Washington USA; ^2^ Fred Hutch, Office of HIV/AIDS Network Coordination Seattle Washington USA; ^3^ Department of Political Science University of California Los Angeles Los Angeles California USA; ^4^ Seattle University, College of Nursing Seattle Washington USA; ^5^ Seattle Children's Center for Pediatric Nursing Research Seattle Washington USA; ^6^ Public Health – Seattle & King County, HIV/STD Program Seattle Washington USA; ^7^ Department of Medicine University of Washington Seattle Washington USA; ^8^ Department of Global Health University of Washington Seattle Washington USA

**Keywords:** community engagement, HIV, informed consent, molecular HIV surveillance, phylogenetics, public health ethics

## Abstract

**Introduction:**

The widespread implementation of molecular HIV surveillance (MHS) has resulted in an increased discussion about the ethical, human rights and public health implications of MHS. We narrate our process of pausing our research that uses data collected through MHS in response to these growing concerns and summarize the key lessons we learned through conversations with community members.

**Methods:**

The original study aimed to describe HIV transmission patterns by age and race/ethnicity among men who have sex with men in King County, Washington, by applying probabilistic phylodynamic modelling methods to HIV‐1 *pol* gene sequences collected through MHS. In September 2020, we paused the publication of this research to conduct community engagement: we held two public‐facing online presentations, met with a national community coalition that included representatives of networks of people living with HIV, and invited two members of this coalition to provide feedback on our manuscript. During each of these meetings, we shared a brief presentation of our methods and findings and explicitly solicited feedback on the perceived public health benefit and potential harm of our analyses and results.

**Results:**

Some community concerns about MHS in public health practice also apply to research using MHS data, namely those related to informed consent, inference of transmission directionality and criminalization. Other critiques were specific to our research study and included feedback about the use of phylogenetic analyses to study assortativity by race/ethnicity and the importance of considering the broader context of stigma and structural racism. We ultimately decided the potential harms of publishing our study—perpetuating racialized stigma about men who have sex with men and eroding the trust between phylogenetics researchers and communities of people living with HIV—outweighed the potential benefits.

**Conclusions:**

HIV phylogenetics research using data collected through MHS data is a powerful scientific technology with the potential to benefit and harm communities of people living with HIV. Addressing criminalization and including people living with HIV in decision‐making processes have the potential to meaningfully address community concerns and strengthen the ethical justification for using MHS data in both research and public health practice. We close with specific opportunities for action and advocacy by researchers.

## 1 INTRODUCTION

HIV surveillance systems are critical for monitoring HIV epidemics, understanding disparities along the HIV care continuum and directing services to communities most impacted by HIV. In the United States, the National HIV Surveillance System includes the collection of partial HIV gene sequences obtained from people living with HIV through routine clinical care for the purposes of drug resistance genotyping. Public health agencies use these sequences to monitor and respond to HIV drug resistance and transmission patterns, a practice called “molecular HIV surveillance” (MHS). As with traditional HIV surveillance data, de‐identified HIV gene sequence data collected through MHS are used in academic research. Phylogenetic analyses of data collected through MHS are a powerful scientific tool due to the high sampling coverage and longitudinally available data made possible through HIV surveillance. MHS data have been used to identify the transmission of multiclass highly drug‐resistant HIV and to characterize the increasing non‐B HIV‐1 subtype diversity within the United States [1, 2]. These types of analyses have important implications for the clinical management of HIV, pre‐exposure prophylaxis efficacy, vaccine development and HIV prevention efforts in local jurisdictions.

Beginning in 2018, the United States Centers for Disease Control and Prevention (CDC) mandated that health departments use HIV gene sequences collected through MHS to identify emerging HIV transmission clusters (defined as people whose HIV sequences have a high degree of genetic similarity) for targeted HIV prevention and linkage‐to‐care efforts. Then, in 2019, cluster detection and response utilizing MHS data were announced as the “fourth pillar” of the Ending the HIV Epidemic initiative [3]. The widespread implementation of MHS for cluster detection and response has resulted in increased discussion about the ethical and human rights implications as well as the public health utility of MHS.

Community concerns highlight issues around consent, stigma, privacy and HIV criminalization that disproportionately impact vulnerable populations of racial and ethnic minorities, people experiencing homelessness, transgender communities, people who inject drugs and sex workers. Although at least eight states have recently modernized their HIV criminalization laws, most of which were written before the availability of antiretroviral therapy and scientific evidence in support of treatment as prevention, 35 states have laws that criminalize the behaviour of people living with HIV [4]. In September 2019, HIV advocates called for a moratorium on all MHS activities until the CDC standardizes data protections, develops partnerships with networks of people living with HIV to facilitate ongoing community engagement and ensures human rights protection [5, 6].

Early discussions of community concerns focused on the use of MHS by health departments with less attention to the use of MHS data for research. In September 2020, the *American Journal of Bioethics* published an issue highlighting the ethics of MHS (Volume 20, Issue 10). In this issue, Molldrem and Smith summarized key bioethical challenges associated with using MHS for public health *as well as* research [7]. These include the criminalization implications of methods that determine the direction of transmission, HIV genotype results obtained without informed consent and the increased stigmatization of communities that disproportionately experience marginalization and oppression.

This rapidly evolving discourse underscores how current MHS practices challenge person‐centred HIV prevention services and research. Person‐centred approaches require that institutions and providers promote individual agency and also recognize how personal, contextual and structural factors—including stigma, racism, and criminalization—impact engagement in HIV prevention and treatment [8, 9]. As argued by Molldrem and Smith, “HIV‐related marginalization means that extra care must be taken by practitioners to ensure not only that HIV data are properly managed, but also that individual and collective rights, personhood, and autonomy are respected during every stage of program implementation” [7].

In this article, we narrate our process of pausing our phylodynamic research that used HIV gene sequences collected through MHS to understand community perspectives about our specific research study. We briefly summarize the original study aims, methods, findings, the key lessons we learned through conversations with community members and advocates, and factors guiding our decision to pause the publication of our study. The authors of this article include the academic research team that conducted phylodynamic analyses, academic researchers conducting concurrent qualitative studies of community concerns related to MHS and community advocates.

## 2 METHODS

### 2.1 Original study

The original study aimed to describe HIV transmission patterns among gay, bisexual and other men who have sex with men in King County, Washington by age, race, and ethnicity. We applied a probabilistic phylodynamic method developed by Volz and Frost [10, 11]. This method uses: (i) a phylogenetic tree reconstructed from HIV‐1 *pol* gene sequences collected through MHS; (ii) person‐level data on CD4 counts and demographics (i.e. age, race/ethnicity); and (iii) a mathematical model of HIV transmission. These inputs were used to estimate the probability of transmission for all pairs in a phylogenetic tree. The resulting pair‐level probabilities cannot reliably determine direct HIV transmission events from one person to another. Rather, when the probabilities are aggregated over a larger population, inferences can be made about population‐level transmission patterns. This method was previously used to understand transmission patterns among men who have sex with men in the United Kingdom [12]. Our analysis estimated: (i) the conditional probabilities associated with one (demographic) group acquiring/transmitting HIV to another group (e.g. younger men had an X% probability of acquiring HIV from older men); (ii) the percentage of excess transmission relative to what was expected under random mixing (e.g. X% excess HIV transmission among Black men); and (iii) Newman's assortativity coefficient.

The original scientific motivations for this analysis were to provide inputs and/or calibration targets to improve the predictive accuracy and specificity of mathematical models that evaluate the impact of interventions on HIV incidence, as well as to inform HIV‐related public health activities in King County. Unlike egocentric surveys, which are time‐ and cost‐intensive and indirectly estimate transmission probabilities via estimates of sexual mixing and dyad‐level behaviours, phylodynamic methods can directly estimate the assortativity parameters.

This research study was part of a set of related HIV molecular epidemiological studies funded by the National Institutes of Allergy and Infectious Diseases. Ethical approval was received from the Washington State and University of Washington (UW) Institutional Review Boards. We employed recommended methods for data security, confidentiality and legal protections [13]: (i) we used de‐identified data and did not have access to identifiable information; (ii) we had formal data and confidentiality agreements with Public Health—Seattle & King County and Washington Department of Health to only use the data for the intended public health‐related research purposes; and (iii) we had a Certificate of Confidentiality (CoC) from the National Institute of Health that allows researchers to refuse to disclose information for civil, criminal, administrative, legislative or other proceedings at the federal, state or local level.

### 2.2 Community engagement

Members of the original study team first became aware of community concerns related to MHS in 2016 [14, 15] and again in early 2019 through a four‐part lecture series facilitated by *The Legacy Project*. Afterwards, we followed the increasing visibility of community concerns related to MHS in the published literature [13, 16], media and conferences. In September 2020, our team read and discussed articles in the *American Journal of Bioethics* [7, 17, 18, 19, 20, 21, 22, 23]. The authors of the original study team (DMT, RPK and JTH) decided to pause the research study to conduct community engagement, specifically due to emerging discussions related to inferring transmission directionality. Due to the analytical method we were applying to MHS data, we anticipated that there would be concerns about the specific phylodynamic method we utilized.

Although there is no single best method for conducting community engagement, it ideally occurs at all stages of the research process, to centre and uplift the experiences of people living with HIV in research that affects their lives. Since we began our community engagement process after analyses were completed, we first consulted with the UW/Fred Hutch Center for AIDS Research Community Engagement Office. We then organized two public‐facing online presentations. The target audience of one presentation was researchers and clinicians (approximately 50 attendees), while the second presentation included local community‐based organizations and HIV advocates (approximately 15 attendees). After we integrated feedback from these sessions into a manuscript draft, we invited two members of a national community coalition on MHS to review and provide feedback on the manuscript. This process included both written and oral feedback given during an hour‐long meeting. Lastly, we held a meeting with eight members of the national community coalition on MHS, which included representatives of networks of people living with HIV across the United States. These activities occurred between October 2020 and June 2021. During each presentation and community consultation meeting, we shared a 10‐ to 15‐minute presentation of our methods and findings that incorporated the feedback we had received to date and explicitly solicited feedback on the perceived public health benefit and potential harm of our analyses and results. The first and senior authors (DMT and JTH) took detailed notes during each meeting, practised reflexive writing and journaling, and met weekly to discuss the issues and topics that arose. The recommendation to share our community engagement process and decision‐making framework for whether to publish the original study was suggested by members of the national community coalition on MHS and was the impetus for this article.

## 3 RESULTS

To provide context for the community feedback we received, we first briefly summarize our original study findings. Our analysis found phylodynamic evidence of assortative transmission by age, race, and ethnicity; that is our modelling indicated that Black, Hispanic/Latino and Asian men were more likely to acquire HIV from men of the same race or ethnicity than would be expected under random mixing. In addition, contrary to prior hypotheses about age‐discrepant HIV transmission, we estimated that younger men acquired HIV from older men less frequently than expected under random mixing.

In the following sections, we summarize the key takeaways from our community engagement exercise. Some community concerns about MHS in public health practice extend to research using MHS data, namely those related to informed consent, inferring directionality and criminalization. We also received critical feedback specific to our research application related to the use of phylogenetic analyses to study assortativity by race and ethnicity, and the importance of considering the broader context of stigma and structural racism.

### 3.1 Informed consent

The lack of informed consent for the future use of HIV biospecimens collected through routine clinical care is central to community concerns about MHS, both in public health practice and its use in research. Historically, public health surveillance programmes have had legal mandates to collect data without informed consent in order to facilitate the collection of complete, timely and reliable data with which to monitor and improve the health and wellbeing of the public [24]. This practice is considered ethically justified in contexts in which public health departments engage impacted communities and the data include the minimum necessary information, are stored securely and used for public health *action* [25]. Some scientists have expressed concern that the collection of informed consent in standard public health surveillance practices may lead to higher rates of refusal for genotypic testing and negatively impact engagement in care [22].

The lack of informed consent in surveillance has also raised ethical issues related to privacy, autonomy and risks/harms posed to vulnerable populations [24, 26]. Importantly, concerns about the lack of informed consent for public health and research reuses of HIV genotype results differ from other forms of public health surveillance due to: (i) the increased risks posed to people living with HIV due to HIV criminalization; and (ii) the unique potential for MHS data, when taken alongside additional epidemiological and clinical data, to infer (and be misinterpreted to prove) specific transmission events. These unique contexts, as well as the influences of stigma and structural racism, are central to understanding why informed consent is a primary community concern about MHS.

A lack of meaningful dialogue around the issue of informed consent has the potential to erode trust in MHS and health systems overall. Throughout our community engagement process, the lack of informed consent for the research use of HIV sequences collected through MHS was described by some individuals as analogous to the harm and distrust created by the Tuskegee Syphilis Study and the HeLa cell line of Henrietta Lacks. Therefore, we believe that progress towards building trust and bridging understanding between researchers, public health agencies and community members will need to meaningfully address concerns related to informed consent and community consultation over the applications of MHS data.

### 3.2 Inferring directionality and criminalization

Community‐based advocacy and scientific publications have increasingly highlighted concerns about the use of HIV sequences to infer the direction of transmission and implications for HIV criminalization cases [7, 27]. As we had anticipated, the phylodynamic method applied in our original study directly touched on these concerns. Although these methods are unable to robustly infer transmission from one individual to another [10], they can be misinterpreted by scientists and community members not familiar with the limitations of phylogenetic methods.

Community members also had concerns that the results of our analysis could be used in HIV criminalization cases. We believe this is not true for our specific study, as our study team would not be required to respond to a subpoena request for the de‐identified MHS data or our phylodynamic results because of the NIH CoC, nor are these data actionable if subpoenaed (as we cannot identify specific individuals). However, these data protections are perhaps not universal or consistent across localities, and not all researchers who possess access to MHS data may be aware of these legal protections. Importantly, this is also not made transparent to the community by researchers and public health agencies. These concerns highlight the different uses of MHS data by research groups compared to public health organizations that *do* have the ability to identify individuals in their cluster detection and response work. Increasing transparency and community engagement around specific uses of MHS data and their implications for criminalization is a key step towards addressing community concerns.

### 3.3 Assortativity, stigma and structural racism

Assortativity, or the increased likelihood of choosing partners from one's own identified subgroup, has been hypothesized to contribute to disparities in HIV incidence [28, 29, 30, 31, 32]. Our original study used data collected through MHS to quantify assortativity by age, race, and ethnicity among men who have sex with men to better understand the role of assortativity in producing disparities. Through our community engagement, we repeatedly heard from people living with HIV and community‐based service providers that these types of analyses felt “voyeuristic,” like “racial profiling,” and that they perpetuated harmful stereotypes. They emphasized that assortative mixing by race and ethnicity occurs in the context of systemic oppression, and in many cases, arises out of the need for in‐group love and support. Therefore, because assortative mixing is not a directly intervenable network‐level characteristic, overemphasizing its role in sustaining disparities has the potential to stigmatize love, sex, healthy coping and partnerships within communities of people of colour.

Although assortative transmission in combination with high community viral load likely contributes to racial and ethnic disparities in HIV, numerous structural and social determinants of health also influence infectious disease transmission dynamics and have been hypothesized to explain these sustained disparities [32, 33, 34, 35, 36]. Structural factors related to racism and stigma play a more fundamental role in producing and sustaining these disparities (and are likely the root structural causes of the sexual network characteristics, like assortativity). For example, HIV criminalization laws, as well as laws that criminalize behaviours (including LGBTQ identities, breastfeeding, sex work, and drug use/possession) disproportionately impact racial minorities and are associated with reduced access to HIV services [37, 38, 39, 40].

Residential segregation is also highly correlated with higher HIV incidence rates among Black populations [33, 41]. There are several pathways through which residential segregation contributes to disparities in HIV. It is an indicator of a concentrated disadvantage as Black families disproportionately live in neighbourhoods with fewer resources and more frequent interactions with a criminal justice system that disproportionally incarcerates people of colour [35, 41]. Residential segregation impacts access to high‐quality healthcare through a lack of local clinics and poor transportation infrastructure [34]. Lastly, higher levels of segregation and socio‐economic inequalities that were present at the onset of the HIV/AIDS crisis of the 1980s contributed to a higher background prevalence of HIV within Black and Latinx communities.

Similarly, stigma likely plays a central role in sustaining racial disparities in HIV. In Earnshaw et al.’s stigma and HIV disparities model, structural racism enacted through residential segregation, historical traumatic assaults and medical distrust can be conceptualized as structural‐level manifestations of stigma. These produce differences in social status and resources that serve to reinforce individual‐level stigma, including prejudice, stereotypes, discrimination and internalized stigma [42].

The role of sexual network characteristics—such as assortativity—as mediators between structural and individual‐level factors has largely been unstudied. Therefore, we recommend that future phylogenetic research aims to better understand how assortativity and transmission patterns are a consequence of structural causes of HIV disparities, including structural racism and stigma.

### 3.4 Deciding to pause publication

Our decision to pause the publication of our original study was guided by the Belmont Report, the Denver Principles, recommendations of the NIH Working Group on Ethical Issues in HIV Phylogenetic Research, and our community consultation process. The 1979 Belmont Report declared three basic ethical principles for research: respect for persons (autonomy), maximize possible benefits and minimize possible harms (beneficence/non‐maleficence) and equitable access to the benefits and burdens of research (justice). The Denver Principles were written by an advisory committee of people with AIDS in 1983. As they relate to people living with HIV's interactions with biomedical institutions, this historic document states the right to “be involved at every level of decision‐making” and “to full explanations of all medical procedures and risks [and] to refuse to participate in research without jeopardizing their treatment” [43]. Lastly, the NIH Working Group discussed the use of public health surveillance data in research, and underscored that due to public health agencies’ “ethical and legal mandate to collect communicable disease data,” research that uses MHS data “must be ethically justified on the basis of potential benefits to public health” [13]. Our community consultation process led us to interpret and apply these principles and recommendations within a new cultural context.

Given that HIV sequences collected through MHS are obtained without informed consent for their future use in research, we felt this raised important questions about the public health utility of our research findings as well as the application of the ethical principles of beneficence/non‐maleficence and justice in this research. Those questions included:

1. Does this research translate into public health action and efforts to end the HIV epidemic? (Public Health Utility)
2. Does this research benefit—or at minimum not harm—communities of people living with HIV who are included in this research without their consent? (Beneficence/non‐maleficence)
3. Does this research equitably distribute benefits and risks to people living with HIV *and* people susceptible to acquiring HIV? (Justice)

Ultimately, guided by the above‐described concerns as well as the lack of *directly* intervenable factors (e.g. assortativity) or public health interventions in the original study, we determined that our original study has insufficiently addressed all three of these questions. Although our community engagement process revealed that some individuals thought our findings could be beneficial for prioritizing the distribution of public health resources and dispelled harmful stereotypes about young men frequently acquiring HIV from older partners, we concluded that these benefits did not balance the potential harms of publishing our study. The two most significant potential harms included (1) perpetuating racialized stigma about/among gay, bisexual and other men who have sex with men and (2) eroding the trust between phylogenetics researchers and communities of people living with HIV by choosing to publish despite their clearly and consistently articulated concerns. Although criminalization was a major theme in community discussions, we believe that our data protections, the inability of our analytical methods to reliably infer transmission directionality and the recent decriminalization of HIV in Washington significantly minimized the potential for harmful outcomes. While we believe research use of MHS data to identify transmission dynamics is an important tool that can inform public health action (even if not directly), we concluded that moving forward with publication requires deeper community engagement and transparency.

## 4 DISCUSSION

The use of MHS data for research—data which are originally collected for routine clinical care and HIV surveillance purposes—raises complex considerations about the role of informed consent and the relationship between public health research and practice. In this article, we described lessons learned from incorporating community engagement at the final stages of a research study in response to growing ethical concerns about MHS and new guidance on conducting ethical HIV phylogenetics research, as well as our decision to not publish our findings [13]. We found that some community concerns about MHS in public health practice also impact research using MHS data, while other critiques were more specific to our research. This was a challenging yet transformative process that required us to practice cultural humility and reflexivity, including ongoing self‐reflection to unpack how our personal experiences, identities and positions as researchers contributed to our lack of awareness regarding community perspectives. While it is uncommon for researchers to publish on their experiences of pausing a study, understanding these types of decision‐making processes may aid other scientists in ethically designing and conducting future research.

Community engagement has long been the cornerstone of ethical HIV research and public health policy. Recent publications have provided guidance for conducting ethical HIV phylogenetics research, highlighting the importance of meaningful community engagement at all stages of the research process and careful consideration of the risks/benefits posed to both individuals and groups of people living with HIV [13, 44]. However, there is also a demonstrable need to directly address ongoing community concerns around the use of MHS data. Structural factors that curtail the human rights of people living with HIV, namely laws and policies that criminalize HIV, are a key barrier to community acceptance of MHS and HIV phylogenetic research more broadly. In line with the CDC and the *Ending the HIV Epidemic* Initiative, which explicitly names HIV criminalization as a structural barrier to HIV prevention, we advocate for states to repeal laws that criminalize HIV [45, 46]. In addition, some researchers may be well‐positioned to facilitate increased dialogue about MHS that meaningfully includes people living with HIV and conduct research examining these issues. Table 1 describes opportunities for researchers to promote autonomy, transparency, and partnerships between communities of people living with HIV, public health agencies and research institutions.

**Table 1:** Areas for advocacy for researchers

**Advocate for structural interventions**: Advocate for state‐level decriminalization of HIV and sex work. Advocate for policies that prohibit the use of MHS data in criminal, civil or immigration investigations or prosecutions. Commit to providing expert testimony in HIV criminal cases and educate courts, judges and lawyers about the limitations of HIV sequence analyses and their inability to reliably infer direct transmission.
**Promote autonomy**: Apply implementation science and community‐based participatory research methods to understand the feasibility of, and barriers to, collecting informed consent as well as the acceptability of alternative methods that promote data justice (e.g. opt‐out systems, data ownership, etc.). Develop partnerships, community advisory boards and other power‐sharing mechanisms with networks of people living with HIV to facilitate ongoing community engagement around the use of MHS data.
**Promote transparency**: Support local, state and national public health agencies to increase transparency. For example, by publicly sharing information about how often HIV gene sequences collected through MHS are analysed, what types of analyses are conducted, what data and confidentiality protections are in place and what potential interventions or contact with people living with HIV may or may not result from these analyses. Assist local, state and national public health departments to evaluate the effectiveness of cluster detection and response (i.e. if this intervention is successful at averting HIV transmission, linking individuals to care, increased HIV testing, etc.). Assist local, state and national public health departments to quantify the cost and financial benefits of MHS systems in response to advocates’ requests to understand the economics of MHS and cluster detection and response.
**Bridge understanding**: Pause research to conduct in‐depth community‐based participatory research and community engagement to determine how to conduct research using data collected through MHS in a way that is acceptable. Facilitate community engagement and restorative justice between community members and public health departments to bridge understanding and build trust around the use of MHS data. Conduct community engagement and rigorous qualitative studies to understand the experiences and perspectives of people living with HIV who have been contacted as a result of cluster detection and response investigations.

### 4.1 Limitations

Our approach had several limitations. Contrary to best practices, our community consultation process occurred after our study analyses were completed. It is possible, had we engaged with the community from the beginning of our study, we would have been able to develop a research question or approach that was acceptable and minimized harm to communities of people living with HIV. In addition, we did not conduct a formal qualitative study, and at the onset of our community engagement process did not anticipate publishing about our process or the key takeaways that emerged from our conversations. The community engagement exercise undertaken by our team represents an initial and ongoing effort to better understand how community concerns about MHS extended to our research. Despite being relatively limited in its scope, our community consultation process was sufficient to aid us in our decision to not publish our original study, and revealed concerns that were consistent with published concerns about MHS. In addition, many of our key takeaways related to informed consent, language, directionality, assortativity, stigma and structural racism may be useful for other researchers.

## 5 CONCLUSIONS

HIV phylogenetics research using data collected through MHS is a powerful scientific technology with the potential to benefit and harm communities of people living with HIV. MHS data can improve our knowledge about HIV and reduce onward transmission of HIV if applied appropriately. It can also increase stigma, distrust and create barriers to HIV prevention and treatment. Due to significant concerns rooted in consent, criminalization and stigma, the benefits of such research need to be clearly outlined and communities should be empowered to set priorities for why, how and when to do such analysis. Addressing criminalization, including people living with HIV in decision‐making processes related to MHS, and increasing transparency have the potential to meaningfully address community concerns and strengthen the ethical justification for the use of MHS data in both research and public health practice.

## COMPETING INTERESTS

DMT received support unrelated to this work from NIH NIAID (grant F31AI152542). BM's role in this work was supported separately with funds from the United States Government Department of Health and Human Services, NIH NIAID, under: (a) Grant 2 UM1 AI068614‐15, “Leadership Group for a Global HIV Vaccine Clinical Trials Network” (Office of HIV/AIDS Network Coordination) to the Fred Hutchinson Cancer Research Center. The authors have no competing interests.

## AUTHORS’ CONTRIBUTIONS

DMT, AS, RPK and JTH conceived of and conducted original studies and analyses. DMT, BM, AT, RPK and JTH participated in the community engagement process as researchers and/or community advocates. DMT drafted the original manuscript, and all authors reviewed, edited and approved the final manuscript.

## FUNDING

This work was supported by the National Institutes of Health (NIH), National Institute of Allergy and Infectious Diseases (NIAID) grant R01AI127232 awarded to JTH and RPK.

## Data Availability

Data sharing is not applicable to this article as no datasets were generated or analysed during the current study.
